# Energy distribution property and energy coding of a structural neural network

**DOI:** 10.3389/fncom.2014.00014

**Published:** 2014-02-21

**Authors:** Ziyin Wang, Rubin Wang

**Affiliations:** Institute for Cognitive Neurodynamics, School of Sicence, East China University of Science and TechnologyShanghai, China

**Keywords:** neural network, nervous energy, neural coding, parameter

## Abstract

Studying neural coding through neural energy is a novel view. In this paper, based on previously proposed single neuron model, the correlation between the energy consumption and the parameters of the cortex networks (amount of neurons, coupling strength, and transform delay) under an oscillational condition were researched. We found that energy distribution varies orderly as these parameters change, and it is closely related to the synchronous oscillation of the neural network. Besides, we compared this method with traditional method of relative coefficient, which shows energy method works equal to or better than the traditional one. It is novel that the synchronous activity and neural network parameters could be researched by assessing energy distribution and consumption. Therefore, the conclusion of this paper will refine the framework of neural coding theory and contribute to our understanding of the coding mechanism of the cerebral cortex. It provides a strong theoretical foundation of a novel neural coding theory—energy coding.

## Introduction

Studying neural coding and decoding is one of the most important, dynamic, and challenging fields of neuroscience (Amari and Nakahara, 2005; Gazzaniga et al., 2009). Yet, there are still large obstacles in researching this problem (Purushothaman and Bradley, 2005; Natarajan et al., 2008; McLaughlin, 2009) because there is no effective theory of neural coding and decoding to investigate global brain activity (Laughlin and Sejnowski, 2003). The practical resources required to solve this problem are insufficient. Apart from technical limitations of experimental neuroscience, most neural computation is confined to several conventional modes based on H-H equations. In the view of neuroscience history, valid theories, and proper assumptions usually guide research to elucidate new phenomena (Abbott, 2008).

The neural activity and operations of the brain involve the principle of depleting minimized energy and maximizing signal transmission efficiency (Laughlin and Sejnowski, 2003). It is logical to exploit these operations to survey the neural coding and cognitive functions of the brain. Indeed, a series of achievements have been obtained by research that uses energy theory to explore the correlation between nervous energy and cognitive behavior (Levy and Baxter, 1996; Raichle and Gusnard, 2002; Wang and Zhang, 2006, 2007, 2012; Wang et al., 2008, 2009). The most remarkable finding is a new functional mechanism of neurons. That is, while firing action potentials, neurons firstly absorb energy, namely oxyhemoglobin, and then consume energy, namely deoxyhemoglobin. This mechanism challenges the traditional energy theory that neurons only consume energy. We found that: (1) action potentials strictly corresponds to nervous energy, and the strict correspondence between neural network activity oscillation and the exiting energy flow. This provides a novel method to investigate neural information processing through nervous energy, which we term neural energy (Wang and Zhang, 2006); (2) The nerve energy encoding can automatically assign the nervous energy corresponding to each frequency of synchronous oscillations in accordance with the allocation ratio in the total energy of the network and separate each neuronal group from the network. This property is significant for functional neural network analysis, especially for higher cognitive functions (Wang and Zhang, 2012); (3) The neural energy can be superimposed, which greatly facilitates the analysis of complex neural networks with large dimensions and strong non-linearity. Moreover, the superimposed energy can reflect the state of synchronous oscillation at the current moment. Synchronous oscillation of cortical processing is closely related to the formation of conscious cognition (Wang and Zhang, 2011); (4) This neural function mechanism can illustrate the biological phenomena that have remained indecipherable so far (Wang and Zhang, 2012), and theoretically demonstrated that neural energy is indeed strongly coupled with variation in blood flow (Moore and Cao, 2008; Lin et al., 2010), which, consequently, provides a theoretical basis for further neural modeling.

The cognitive and conscious activity of brain actually functions through control of the transition between structural networks and cognitive networks. It is necessary to study a systematical method which addresses how the neural computing of local network relate to the overall mode of neural activity. Synchronous oscillation which caused by dynamic equilibrium among different regions and functional combination originates from polyrhythmic motions of neural populations in encephalic regions. This paper is preliminary and basic prob into cognitive neurodynamical mechanism of synchronous oscillation from a novel aspect.

Yet, this method it is confined to the structural connection of neural networks rather than functional connection in research with the energy method (Wang and Wang, under review). Though it is an important issue, there are no existing reports regarding neural energy coding in the cerebral cortex and how structural connections convert to functional connection. The aim of this paper is to investigate the energy-coding mode of structural networks by adjusting the networks' parameters based on structural connections. It may provide strict theoretical bases for further research about networks' energy derivation and their corresponding coding mode during the conversion from structural to functional connections.

Nervous energy is related with neural signal coding (Wang and Zhang, 2006, 2007; Wang and Wang, under review), and network behavior is also inherently correlated to the parameters. Therefore, the total energy caused by the overall network activity is interwoven with its parameters (Dhamala et al., 2004). In other words, network activity and behavior controlled by parameters (Ghosh et al., 2008), e.g., neuron number, coupling strength, and the transform delay, can be described by the network energy as it is in oscillation.

Previously, we showed that as a neuron fires which occur the action potential, it firstly discharges the energy in storage (negative energy), and then oxyhemoglobin supplies additional energy (Wang and Zhang, 2012; Wang and Wang, under review). The characteristic about negative energy can effectively reflect neuronal synchronization. In this paper, this property is taken further to investigate the correlation between network parameters and an energy-related index, which is the ratio of discharged energy to accumulatively consumed energy.

## Biophysical model of neurons and their energy function

**Figure 1:**
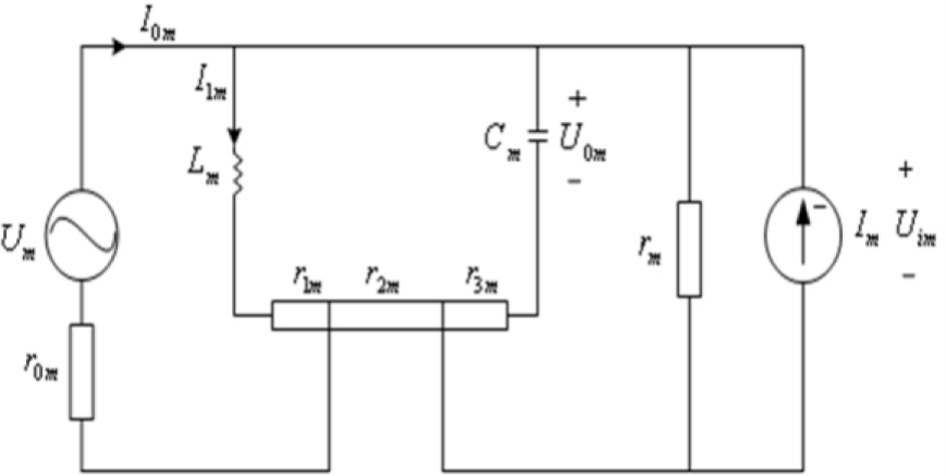
**Biophysical model of a neuron**.

In order to simulate the energy neuron network coding, we propose a novel biophysical model of a neuron comprised of voltage and current sources and an inductor (Figure 1). The voltage source is the difference in concentrations of various extra- and intracellular ions that induce ion movement. The current source is formed by ion concentration gradients and the stimulated by neighboring neurons. Moreover, when charged ions (*K*^+^, *N*^+^_*a*_, *C*^+2^_*a*_) flow into and out of the ion channels, a self-induced loop current is formed, which is equivalent to a voltage source. The current and the voltage sources can cause energy loss, *r*_*m*_ and *r*_0*m*_ describe the resistance across $P=\sum_{m\;=\;1}^{N}P_{m}$ and *U*, respectively. The current and voltage sources are not affected in the same position, and film resistor can be divided by *r*_1*m*_, *r*_2*m*_, and *r*_3*m*_. The sum of the input current from connected neurons describes the coupling relation:

(1) $$\begin{array}{l} I_{m}=i_{1m}+\sum_{j=1}^{n}\left[i_{0m}(j-1)\sin(\omega_{m}(j-1)(t_{j}-t_{j-1}))\right]\\ \;+i_{0m}(n)\sin(\omega_{m}(n)(t-t_{n})) \end{array}$$

Where *i*_*m*1_ is the current to maintain the resting membrane potential, *i*_0*m*_ is the total effect generated by the current stimulation of peripheral neurons, and ω_*m*_ is firing frequency.

The circuit equation corresponds to Figure 1:

(2) $$\{\begin{array}{l} U_{m}=r_{0m}I_{0m}+r_{1m}I_{1m}+L_{m}{\dot{I}}_{1m}\\ I_{0m}=I_{1m}-I_{m}+\frac{U_{im}}{r_{m}}+C_{m}{\dot{U}}_{0m}\\ U_{im}=C_{m}r_{3m}{\dot{U}}_{0m}+U_{0m} \end{array}$$

and

(3) $$L_{m}{\dot{I}}_{1m}+r_{1m}I_{m}=K_{1m}{\dot{U}}_{0m}+K_{2m}U_{0m}-r_{2m}I_{m},$$

here $K_{1m}=C_{m}(r_{2m}+r_{3m}+\frac{r_{2m}r_{3m}}{r_{m}})$, $k_{2m}=1+\frac{r_{2m}}{r_{m}}$

The total power consumed by the neural network composed of *N* neurons is

(4) $$P=\sum_{m=1}^{N}P_{m}$$

where the power consumed by the *m*th neuron is

(5) $$\begin{array}{l} P_{m}=d_{1m}{\dot{U}}_{0m}^{2}+d_{2m}{\dot{U}}_{0m}+d_{3m}{\dot{U}}_{0m}U_{0m}+d_{4m}U_{0m}^{2}\\ \;+d_{5m}U_{0m}+d_{6m} \end{array}$$

here *d*_1*m*_, *d*_2*m*_, *d*_3*m*_, *d*_4*m*_, *d*_5*m*_, and *d*_6*m*_ are the same as in Wang and Zhang (2011).

According to the theory of circuit, energy is the integral of power. All the “power” in our subsequent simulations are instantaneous power. In this sense, the power of neuron and the energy of neuron is consistent concepts.

According to Equations (2)–(5), we obtain

(6) $$\begin{array}{l} U_{0m}=-\frac{{\hat{g}}_{1}}{\lambda_{m}^{2}}-\frac{{\hat{g}}_{2}e^{-a(t-t_{n})}}{\lambda_{m}^{2}-a^{2}}-\frac{1}{\lambda_{m}^{2}+\omega_{m}^{2}}({\hat{g}}_{3}\sin(\omega_{m}(n)(t-t_{n}))\\ \;+{\hat{g}}_{4}\cos(\omega_{m}(n)(t-t_{n}))(U_{0m}(t_{n})+\frac{{\hat{g}}_{1}}{\lambda_{m}^{2}}+\frac{{\hat{g}}_{2}}{\lambda_{m}^{2}-a^{2}}\\ \;+\frac{{\hat{g}}_{4}}{\lambda_{m}^{2}+\omega_{m}^{2}(n)}))e^{-\lambda_{m}(t-t_{n})} \end{array}$$

**Figure 2:**
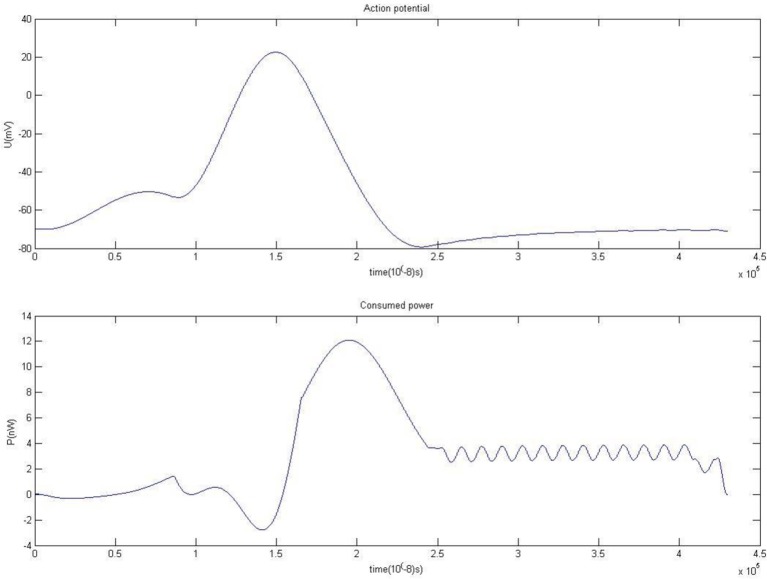
**Action potential and energy function**.

The simulation result of membrane potential and power consumed during firing is shown in Figure 2. This novel biophysical model can simulate neuronal firing well. Because there are the inertia-stored energy elements of capacitance *C*_*m*_ and inductance *L*_*m*_, the peak of consumption curve occurs later than the membrane potential. The inertia-stored energy elements in the biophysical model and the corresponding solving process is described in detail in Wang and Zhang (2007), Wang et al. (2009).

$$\begin{array}{l} r_{0m}=0.0001\;\Omega ,r_{1m}=0.1\;\Omega ,r_{2m}=1000\;\Omega ,r_{3m}=0.1\;\Omega ,\\ r_{m}=1000\;\Omega ,C_{m}=1\;\mu F,L_{m}=50\;\mu H,i_{0m}=70.7\;\mu A \end{array}$$

The positive and negative parts in the power curve may explain why cerebral blood flow is substantially increased whereas oxygen consumption is only slightly increased when neurons are activated. The shape of the power curve may also explain why there is a synchronous effect between the external stimulus and perception generation (Igarashi et al., 2007; Wang and Zhang, 2012), which is still an otherwise inexplicable neurophysiological phenomenon (Dhamala et al., 2004; Haken, 2007; Wang and Zhang, 2012).

The decrease in energy consumption, especially the period of negative energy consumption, is primarily due to the local hyperemia induced by neural activity. Blood flow is increased by dilatation of blood vessels, which augments arterial blood flow and the local oxyhemoglobin content of brain tissue. Neurons absorb oxygen from oxyhemoglobin, but the oxygen consumption does not rise proportionately with this increased blood flow and oxygen supply on the millisecond timescale. Indeed, Fox et al. (Lin et al., 2010) observed that the oxygen extraction fraction (OEF) decreased from a resting value of approximately 40% to approximately 20% during activity associated with task performance. That is, the majority of oxygen carried in oxyhemoglobin is not metabolized by neurons, and only a small number of oxygen is absorbed in neurons. So the overall result is that neurons first exploit energy from the blood after the stimulus, which manifests the negative consumption phase.

In terms of the relationship between energy and cognitive behavior, one important example is Chengyu Li's research (Li et al., 2009). This paper points out that cluster firing of neurons in cerebral cortex can shift two behavioral status of sleep and wakefulness. Subthreshold and supthreshold firing states are closely related to neural energy. We need to stress that the energy analysis of structural neural networks of this paper is the basis of further analysis for functional neural networks, which closely related to higher cognitive function.

## Structural neuronal networks model

**Figure 3:**
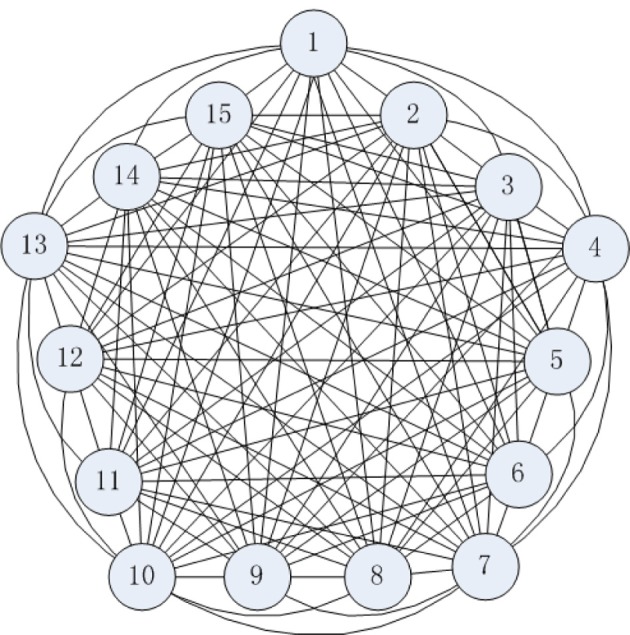
**Neural network connection structure**.

The connection structure of a cortical neural network is shown as Figure 3, where a neuron couples with all other neurons in the model. The characteristics of each neuron are represented by the biophysical model (Figure 1). Therefore, the following neural network structure is strictly defined on a neurobiological basis (Wang and Zhang, 2006, 2007).

The connections among cortical neurons are complicated, one neuron couples with an estimated 10^4^ peripheral neurons (Huang, 2000; Singer, 2009). The global-connected neural network is composed of 15 neurons in Figure 3 in order to understand the energy-coding mode in the stimulated neural network. The neurons are coupled with bidirectional asymmetry coupling strengths. For example, the 1th neuron is coupled to the 2th neuron with coupling strength 0.15, and the 2th neuron is coupled to 1th neuron with coupling strength 0.22. A synaptic coupling strength between neurons is uniformly distributed in the statistical sense (Rubinov et al., 2011), and we assume that a synaptic coupling strength is uniformly distributed in [0.1, 0.3].

Let the coupling strength matrix:

$$W=\left[\begin{array}{cccc} w_{1,\;1} & w_{1,2} & \ldots & w_{1,n}\\ w_{2,1} & \ddots & & w_{2,n}\\ \vdots & & \ddots & \vdots \\ w_{n,1} & w_{n,2} & \ldots & w_{n,n} \end{array}\right]$$

*w*_*i, j*_ is coupling strength when the *i*th is coupled to *j*th.

The network operates as follows:

(7) $$\begin{array}{l} S_{j}(t)=W\times Q{\left(t-\tau \right)}^{T}\\ I_{m,\;j}(t)=\{\begin{array}{l} i_{m1}+\sum_{j\;=\;1}^{n}\begin{array}{l} \begin{array}{c} \left[i_{0m}\left(j-1\right)\sin(\omega_{m}(j-1\right)\\ \left(t_{j}-t_{j\;-\;1}))\right] \end{array}\\ {}[3pt]+i_{0m}(n)\sin(\omega_{m}(n)(t-t_{n}))\text{ if }S_{j}(t)>\text{th} \end{array}\\ i_{m1}\text{ if }S_{j}(t)<\text{th} \end{array} \end{array}$$

Substituting *I*_*m, j*_(*t*) into Equation (5), we obtain *U*_0*m, j*_(*t*).

Substituting Equation (5) into Equation (4), we obtain *P*_*m, j*_(*t*), where *S*_*j*_(*t*) is the sum of the stimulations for the *j*th neuron at time *t*; *Q*(*t* − τ) = [*Q*_1_ (*t* − τ), *Q*_2_ (*t* − τ),…, *Q*_*j*_ (*t* − τ),… *Q*_*n*_ (*t* − τ)] indicates neuron-firing states, which take values of 0 at resting and 1 at firing.

## Power-consuming property during oscillation in various parameters

### The energy-consuming property of neural network under instantaneous stimulus

**Figure 4:**
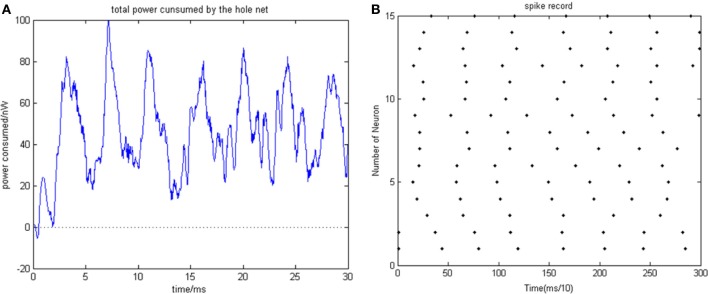
**(A)** Total energy consumed by the overall network with 15 neurons under the condition of instantaneous stimulation. **(B)** Overall network firing record with 15 neurons under the condition of instantaneous stimulation (the coupling strength is uniformly distributed in [0, 1]; the transform delay of synapsis distributed in [0.3 ms, 0.18 ms]).

Given the biological neural network model of 15 neurons, the total energy consumed by the overall network and the firing record under the condition of instantaneous stimulation is shown in Figures 4A,B.

In Figure 4, the 1st and 2nd neuron are instantaneously stimulated with −40 mV strength for 0.1 ms. These two neurons consequently fired under the stimulation, which caused the subsequent cascade reaction. Figure 4A shows the total power consumed by the overall network in 30-ms simulation time. Where the coupling strength is uniformly distributed in [0, 1]; the transform delay of synapses is uniformly distributed in [0.3 ms, 0.18 ms]. The neural impulse is record in Figure 4B, the black dot at coordinate (*t, j*) represents neuron *j* firing a neural impulse at the moment of *t*. Accordingly, the more streak-like the recording is, and the narrower the streak is, the higher the synchronism of the network behavior.

According to Figure 4, the total energy-consuming curve shows no prominent periodicity over time. The negative energy existed at the beginning of the oscillation but was not absorbed subsequently. The simulation result of Figure 4 apparently shows that the oscillation of the network is not synchronous. In order to quantitatively estimate the synchronicity of the network activity under instantaneous stimulation, the mean-max correlation coefficient (MCC) was employed. It is well-known that correlation coefficient is used to determine the degree of synchronicity. Given a biological neural network that consists of *n* neurons, a *n* × *n* correlation coefficient matrix can be calculated. Let this matrix be *C*, and the element at *i*th row and *j*th column be *c*_*ij*_, hence *c*_*ij*_ stands for the correlation coefficient of *i*th and *j*th neurons' membrane potentials. Previous research indicates that once the network synchronized, there will be at least two oscillating groups in the steady state. As the first neural group fires, other groups are stimulated. The simulation is accepted after a tiny interval at the presence of transform delay, then the stimulated groups consequently fire, while the first group came to rest at this period and is re-simulated by the currently firing group. In this way, a coupling oscillation is formed. Therefore, the membrane potential of a neuron *i* is different from other *n* − 1 neurons, we choose the max correlation coefficient (except the correlation coefficient between the neuron's membrane potential and itself where *c*_*ii*_ = 1) to outline the synchronization degree of the neural group containing neuron *i*. Let this the max correlation coefficient of neuron *i* be ρ^*i*^_max_. Accordingly, an average value $\frac{1}{n}\sum_{i\;=\;1}^{n}\rho_{{}_{\max}}^{{}^{i}}$ can be yielded from ρ^1^_max_, …, ρ^*n*^_max_.

The MCC is defined as follow:

(8) $$\rho_{\text{mean}}=\frac{\sum_{i=1}^{N}\max(C_{i,1},C_{i,1},\ldots C_{i,j},\ldots ,C_{i,n})}{N}\begin{array}{c} \;(i\;\neq \;j) \end{array}$$

Where *C*_*i, j*_ is the correlation coefficient between the membrane potentials of neuron *i* and *j*, namely:

(9) $$C_{i,j}=\frac{\sum_{k=1}^{T/\Delta t}\left|(V_{i}(k\Delta t)-\bar{V_{i}})||V_{j}(k\Delta t)-\bar{V_{j}}\right|}{\sqrt{\sum_{k=1}^{T/\Delta t}{\left(V_{i}(k\Delta t)\;-\;\bar{V_{i}}\right)}^{2}{\left(V_{j}(k\Delta t)-\bar{V_{j}}\right)}^{2}}}$$

Note that the result of numerical simulation is a discrete sequence over time. In Equation (9), Δ*t* stands for the sampling interval, and *V*_*i*_ stands for the mean value of the *i*th neuron's membrane potential. It is obvious that as the correlation coefficient between two neurons approaches 1, the stronger the degree of the synchronization is.

**Figure 5:**
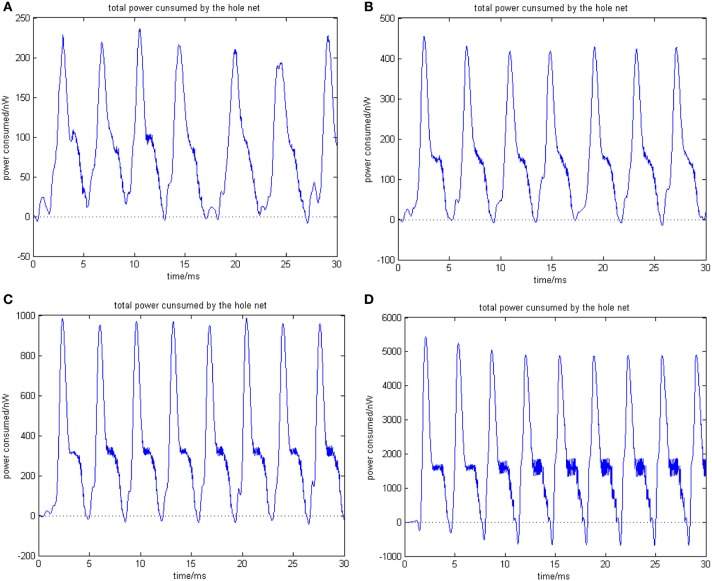
**(A)** The network consists of 30 neurons, ρ_mean_(*a*) = 0.76423. **(B)** The network consists of 50 neurons, ρ_mean_(*b*) = 0.8555 (the coupling strength is uniformly distributed in [0, 1]; the transform delay of synapsis distributed in [0.3 ms, 0.18 ms]). **(C)** The network consists of 100 neurons, ρ_mean_(*c*) = 0.9476. **(D)** The network consists of 500 neurons, ρ_mean_(*d*) = 0.9998 (the coupling strength is uniformly distributed in [0, 1]; the transform delay of synapsis distributed in [0.3 ms, 0.18 ms]).

**Figure 6:**
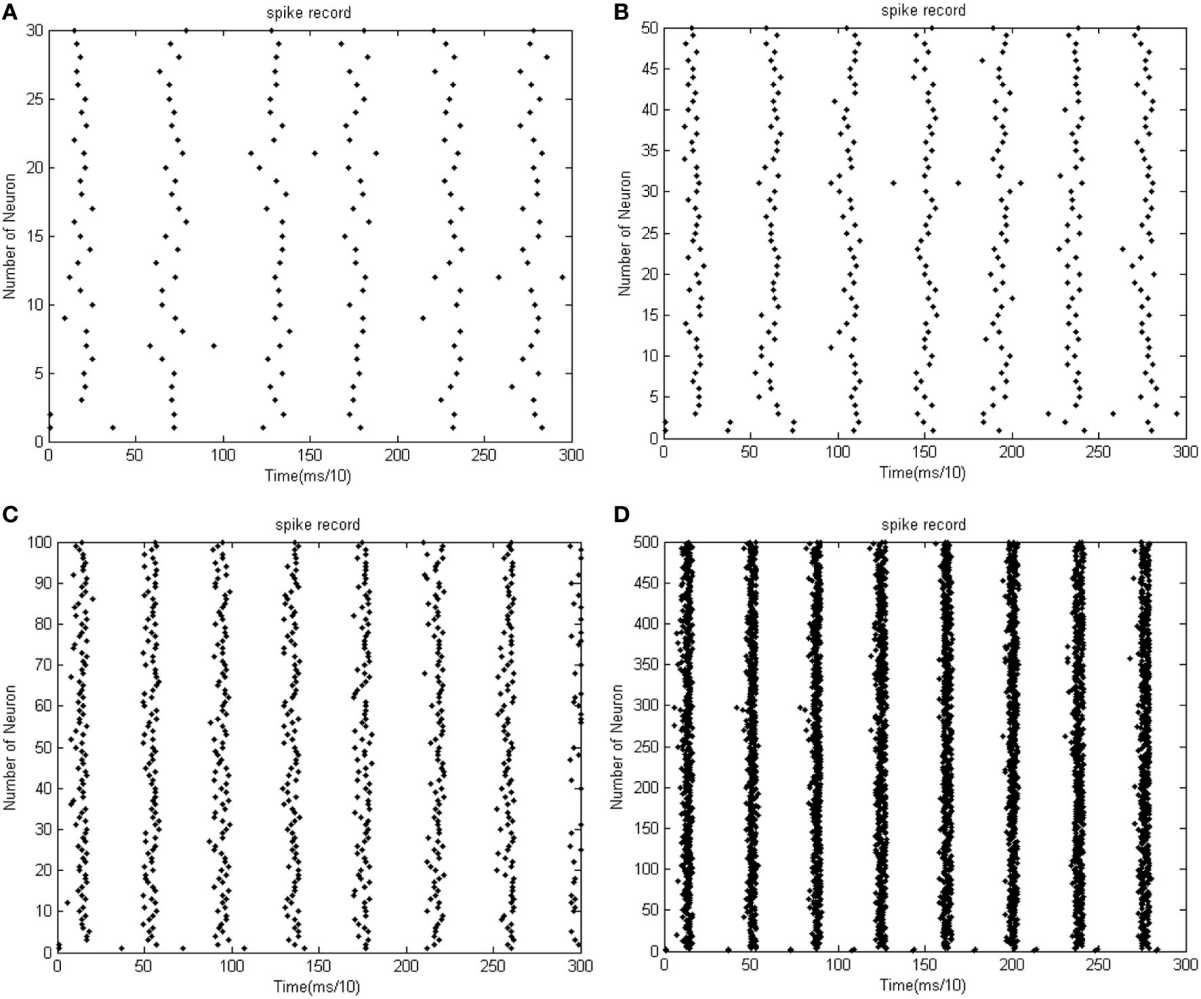
**(A)** The firing record for network with 30 neurons. **(B)** The firing record for network with 50 neurons (the coupling strength is uniformly distributed in [0, 1]; the transform delay of synapsis distributed in [0.3 ms, 0.18 ms]). **(C)** The firing record for network with 100 neurons. **(D)** The firing record for network with 500 neurons (the coupling strength is uniformly distributed in [0, 1]; the transform delay of synapsis distributed in [0.3 ms, 0.18 ms]).

The correlation coefficients among neurons can deal with both partial synchronization and coexisting synchronization in multiple clusters. For each neuron, we choose the maximum of correlation coefficients with other neurons, and then calculate the mean value of these maximums. The approaching to 1 of ρ_mean_ denotes that network's behavior is approaching the state of coexisting synchronization in multiple clusters; on the other hand, the approaching *t* 0 of ρ_mean_ denotes that only a subset of neurons are in a synchronized state, which means no coexisting synchronization in multiple clusters in a network's behavior. In Figure 4 ρ_mean_ = 0.58145, which is in accordance with the firing record showing that no salient synchronization could be drawn. Figures 5A–D shows the total energy consumed by the overall network in 30-ms simulation time when the neuron number is increased. The firing records are shown in Figures 6A–D.

In Figure 5, the 1st and 2nd neuron are stimulated at the moment *t* = 0. The stimulation intensity is −40 mV, and the stimulation time is 0.1 ms, thus leading the stimulated neurons to fire action potentials. The energy consumed by the overall network is recorded, the coupling strength is uniformly distributed in [0, 1], and the transform delay of synapses is distributed in [0.3 ms, 0.18 ms].

Figure 5 shows that as the number of neurons increases, the periodicity becomes salient, and a negative energy component emerges under the situation that coupling strength and transform delay remain steady. Meanwhile, the synchronous motion of the neural firing frequency tends to be prominent as the number of neurons increases.

For facilitation of quantitative analysis, an index is proposed to outline the characteristics of energy distribution: negative energy ratio α(*t*). It is defined as the specific value in the absolute value of negative energy and the sum of the absolute value of negative and positive energy during the time period form moment 0 to moment *t*. That is,

(10) $$\alpha (t)=\frac{E_{\text{negative}}}{E_{\text{positive}}}\times 100\%$$

(11) $$E_{\text{negtive}}=\sum_{i=1}^{n}\int_{o}^{t}P_{i}(t)\times sgn(-P_{i}(t))dt$$

(12) $$E_{\text{positive}}=\sum_{i=1}^{n}\int_{o}^{t}P_{i}(t)\times sgn(P_{i}(t))dt$$

Where *P*_*i*_ (*t*) is the power consumed by neuron *i* at *t* moment, and the integration of *P*_*i*_(*t*) in [0, *t*] stands for the energy consumed in this period. *sgn*(·) is signal function, which is defined as $sgn(x)=\{\begin{array}{l} 1,\begin{array}{cc} & x>0 \end{array}\\ 0,\begin{array}{cc} & x\leq 0 \end{array} \end{array}$. α(*t*) is a foundation of neural energy analysis. The corresponding negative ratio and MCC for Figures 5A,B is shown in Table 1.

**Table 1:** **The corresponding negative ratio and mean-max correlation coefficient for Figures 5A,B**.

**Neuron amount**	**30**	**50**	**100**	**500**
α(*t*) (%)	0.1526	0.2362	0.4532	1.9457
ρ_mean_	0.7642	0.8556	0.9746	0.9998

The distribution interval of couple strength: [0, 1]

The distribution interval of transform delay [0.3 ms, 1.8 ms].

According to the simulation results of Figures 5, 6 and Table 1, the firing recording tends to be streak-like as the number of neurons increases, and greater streak width indicates that the synchronization tends to strengthen the oscillation. The numerical calculation in Figures 5A–D shows that the corresponding MCC efficiency increases. The negative energy ratio is also monotonously increased with the number of neurons. In addition, the energy distribution outlined by the power curve is closely related to network synchronization. Figures 5, 6 demonstrate that this correlation is monotonic. That is to say, in a coupled neuron network, a larger number of neurons makes it easier for the network to synchronously oscillate, and, simultaneously, the negative energy ratio increases (energy is the integration of power, in Figure 5, it is the area cut by power curve and the x-axis).

Figures 5D, 6D show 500 neurons in synchronous oscillation; the periodicity of power consumed is remarkably salient in synchronous oscillation, and the negative energy power increases prominently. The streak of neural firing recording is slender and dark feature. The period form moment 0 to 14 ms is a transient process: the peak of power was lower, and the negative energy augmented this feature. After 14 ms, the energy distribution presented prominent periodicity, suggesting that the network achieved a synchronous oscillation state.

### Correlation between coupling strength and energy distribution

**Figure 7:**
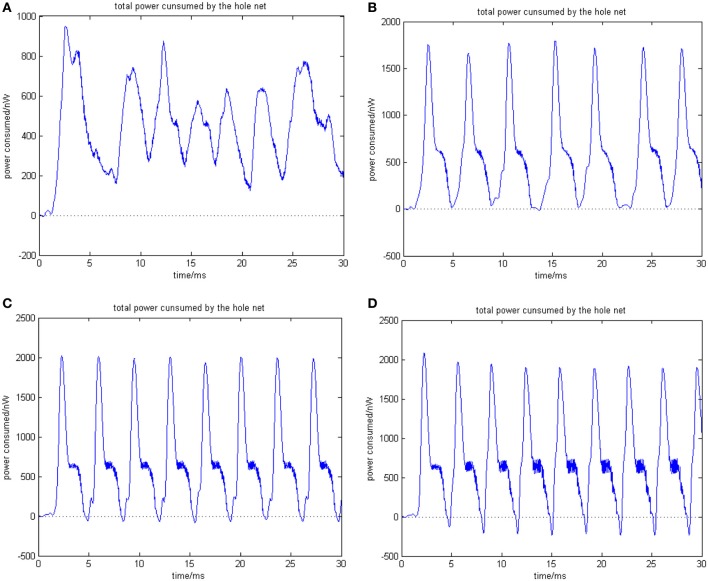
**(A)** Total power consumed by the overall network the coupling strength is uniformly distributed in [0, 0.01] ρ_mean_ = 0.8567. **(B)** Total power consumed by the overall network the coupling strength is uniformly distributed in [0, 0.1] ρ_mean_ = 0.8567. The amount of neuron is 200, and the transform delay is uniformly distributed in [0.3 ms, 1.8 ms]. **(C)** Total power consumed by the overall network the coupling strength is uniformly distributed in [0, 0.05] ρ_mean_ = 0.9470. **(D)** Total power consumed by the overall network the coupling strength is uniformly distributed in [0, 1] ρ_mean_ = 0.9827. The amount of neuron is 200, and the transform delay is uniformly distributed in [0.3 ms, 1.8 ms].

**Figure 8:**
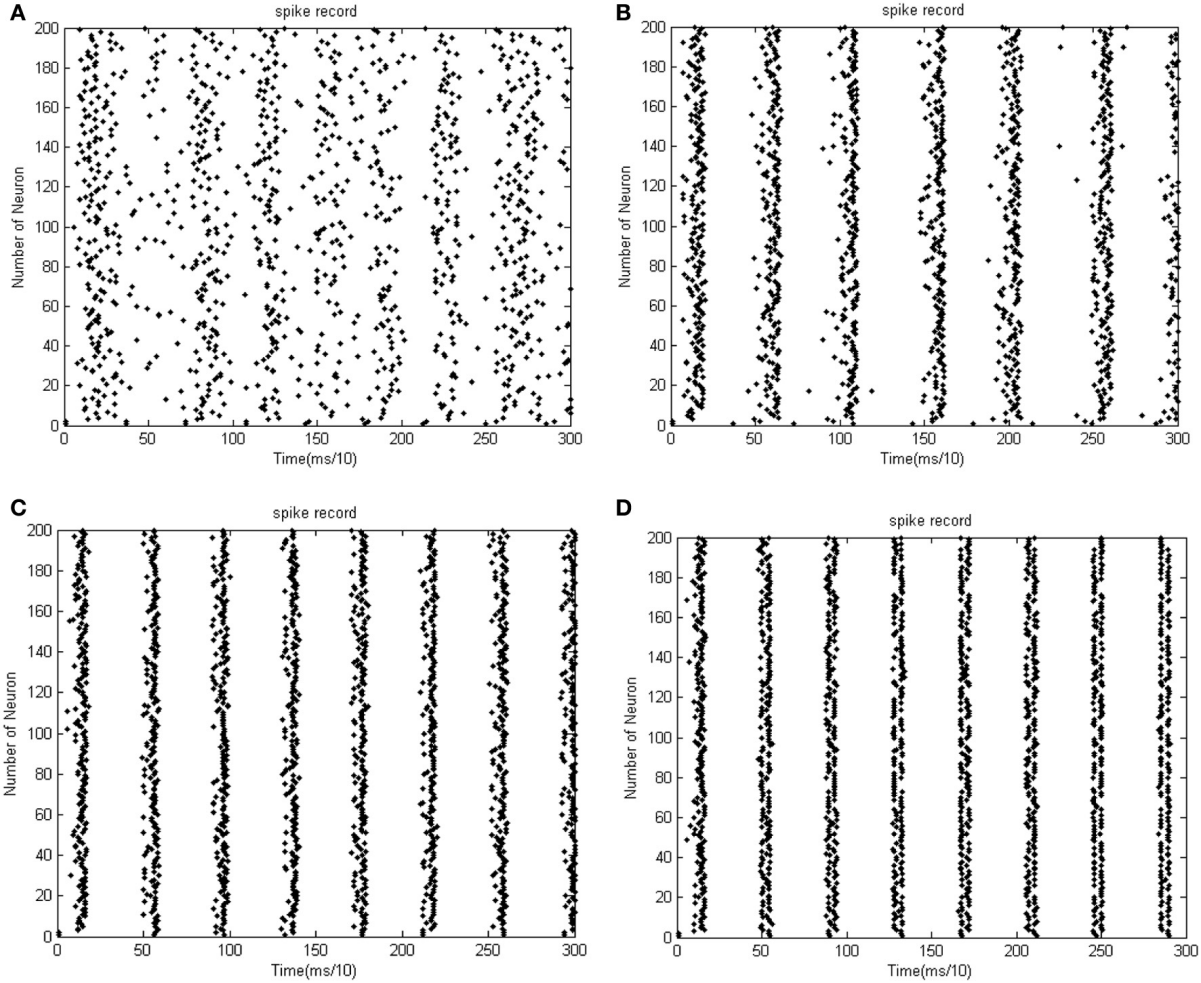
**(A)** The firing impulse record of the network's neural activity the coupling strength is uniformly distributed in [0, 0.01]. **(B)** The firing impulse record of the network's neural activity. The coupling strength is uniformly distributed in [0, 0.1]. The amount of neuron is 200, and the transform delay is uniformly distributed in [0.3 ms, 1.8 ms]. **(C)** The firing impulse record of the network's neural activity the coupling strength is uniformly distributed in [0, 0.01]. **(D)** The firing impulse record of the network's neural activity the coupling strength is uniformly distributed in [0, 0.1]. The amount of neuron is 200, and the transform delay is uniformly distributed in [0.3 ms, 1.8 ms].

Given a neural network comprised of 200 neurons, the transform delay is uniformly distributed in [0.3 ms, 1.8 ms]. Figures 7A–D shows the total power consumed by the overall network when the coupling strength is distributed in [0, 0.01], [0, 0.1], [0, 0.5], and [0, 1], respectively. The firing record during oscillation is shown in Figures 8A–D.

In the simulation of Figures 7, 8, the 1st and 2nd neurons are stimulated at the moment *t* = 0, the stimulating intensity is −40 mV, and the stimulation lasted 0.1 ms. In order to avoid overall tranquillization (where the coupling strength between the stimulated neurons and the others is so slight that other neurons cannot be stimulated to fired), the coupling strength between the stimulated neurons (1st and 2nd neuron) maintained a uniform distribution in [0, 1]. At the same time, coupling strength among other neurons is uniformly distributed in [0, 0.01], [0, 0.1], [0, 0.5], and [0, 1], respectively, for Figures 7A–D. The corresponding negative energy ratio and MCC are displayed in Table 2.

**Table 2:** **The corresponding negative energy and mean-max correlation coefficient**.

**Coupling strength**	**[0, 0.01]**	**[0, 0.1]**	**[0, 0.5]**	**[0, 1]**
α(*t*) (%)	6.5122 × 10^−3^	1.0094 × 10^−2^	0.4919	1.4588
*ρ*_mean_	0.8567	0.9332	0.9470	0.9827

The amount of neurons: 200.

Distribution Interval (uniform) [0.3 ms, 1.8 ms].

According to the calculations of Figures 7, 8, and Table 2, as the coupling strength increases, the periodicity, negative energy ratio, and MCC of the total power curve and the synchronization reflected by firing recordings exhibited a consonant monotonicity. In other words, the total power curve usually appears to be approximately periodic when the oscillations reach a steady state, and the larger values of negative energy ratio and MCC and the salience of synchronization are reflected by the firing recordings.

### Correlation between excitable signal transform delay and energy distribution feature

**Figure 9:**
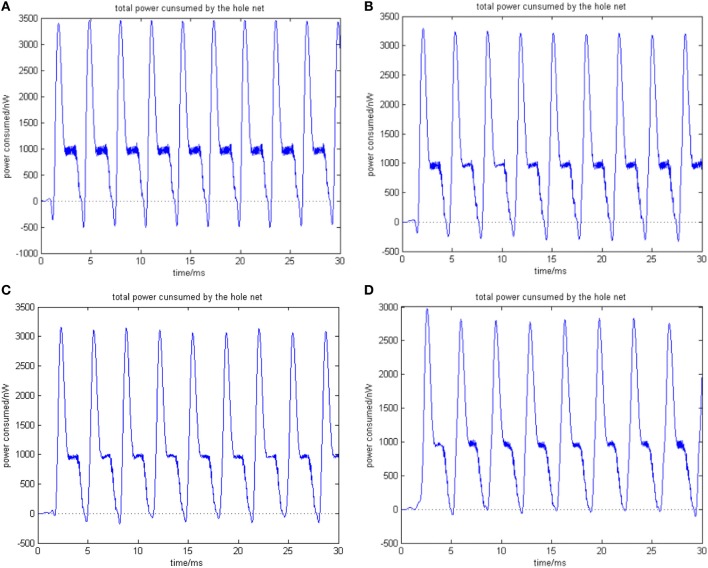
**(A)** Signal transform delay is uniformly distributed in [0.1 ms, 1.6 ms] ρ_mean_ = 0.9980. **(B)** Signal transform delay is uniformly distributed in [0.2 ms, 1.7 ms] ρ_mean_ = 0.9982. The amount of neuron is 300, and the coupling strength is uniformly distribute in [0, 1]. **(C)** Signal transform delay is uniformly distributed in [0.3 ms, 1.8 ms] ρ_mean_ = 0.9980. **(D)** Signal transform delay is uniformly distributed in [0.5 ms, 2 ms] ρ_mean_ = 0.9982. The amount of neuron is 300, and the coupling strength is uniformly distribute in [0, 1].

**Figure 10:**
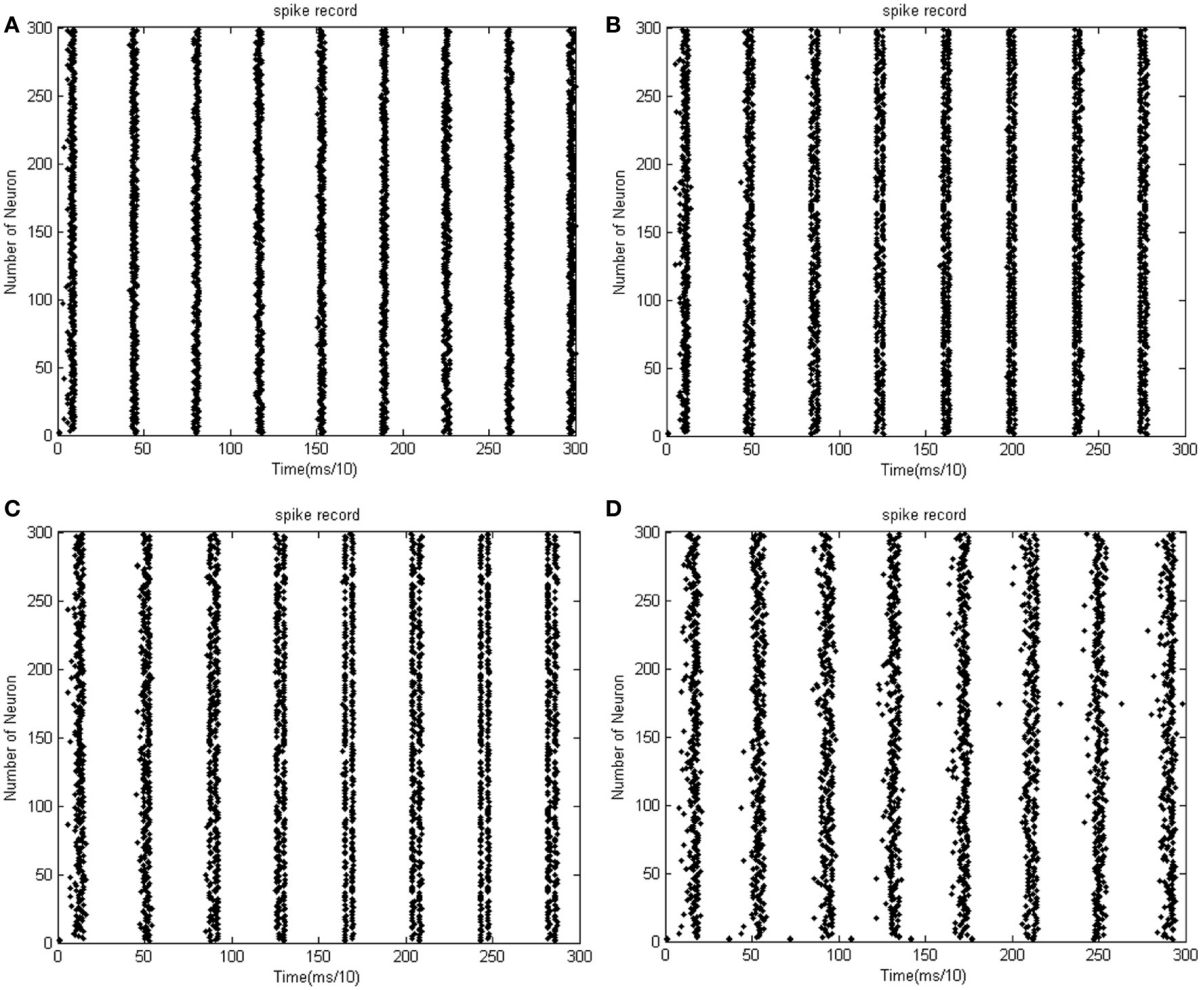
**(A)** The firing impulse record of the network's neural activity signal transform delay is uniformly distributed in [0.1 ms, 1.6 ms]. **(B)** The firing impulse record of the network's neural activity signal transform delay is uniformly distributed in [0.2 ms, 1.7 ms]. The amount of neuron is 300, and the coupling strength is uniformly distribute in [0, 1]. **(C)** The firing impulse record of the network's neural activity signal transform delay is uniformly distributed in [0.3 ms, 1.8 ms]. **(D)** The firing impulse record of the network's neural activity signal transform delay is uniformly distributed in [0.5 ms, 2 ms]. The amount of neuron is 300, and the coupling strength is uniformly distribute in [0, 1].

Given the number of neurons and the constant distribution interval of coupling strength, the feature of total energy consumed by the overall network varies as the signal transform delay changes. Figures 9A–D shows that the total power consumed by the overall network as the distribution interval of signal varies under the condition that the number of neurons is maintained at 300, and the coupling strength is uniformly distributed in [0, 1]. Figures 10A–D shows the corresponding firing impulse recordings during the oscillation.

In the simulation of Figures 9, 10, the 1st and 2nd neuron are stimulated at the moment *t* = 0 at −40 mV, and the stimulation lasted 0.1 ms. The coupling strength is uniformly distributed in [0, 1] while the signal transform delay is uniformly distributed in [0.1 ms, 1.6 ms], [0.2 ms, 1.7 ms], [0.3 ms, 1.8 ms], and [0.5 ms, 2 ms], respectively. The corresponding negative energy ratio and MCC are displayed in Table 3.

**Table 3:** **The corresponding negative energy and mean-max correlation coefficient**.

**The distribution interval of transform delay**	**[0.1 ms, 1.6 ms]**	**[0.2 ms, 1.7 ms]**	**[0.3 ms, 1.8 ms]**	**[0.5 ms, 2 ms]**
α(*t*) (%)	2.731	1.7609	1.6832	0.6131
*ρ*_mean_	0.9980	0.9982	0.9986	0.9671

The amount of neurons: 300.

The distribution interval of [0, 1].

According to Figure 9 and Table 3, the negative energy ratio increases as the distribution interval of transform delay approaches zero. The direction of the correlation coefficient, however, cannot guide any persuasive conclusions. This is because the MCC merely reflects the local synchronization among one specific neural population. As multi-population oscillation is formed under the instantaneous stimulation, the intergroup synchronization cannot be reflected by MCC. According to the neural impulse recorded in Figure 10, it is obvious to note that the intergroup synchronization decreases as the distribution interval of transform delay is further from zero. In Figure 10C, it can be easily perceived that in two stripes with a relative wider interval, called a first-order stripe, exist two stripes with a relative narrow interval, called a second-order stripe, which means that two “micro” oscillation groups exist in each “macro” oscillation group. Because the synchronous degree between the micro oscillation group correspondence to the second-order stripe is high, no definitive result can be achieved by quantifying the correlation coefficient. With regard to thee measure of neural energy, however, the intergroup non-synchronization can be effectively clarified by the negative energy ratio.

The reason why energy analysis can quantitatively analyze the intergroup synchronization is that the negative power only emerges during a tiny period at the beginning of the neural action process, and it takes a trivial ratio of the total energy. If the power of neurons is linearly superimposed, the negative component will be offset by the positive component when the oscillation synchronization is not salient, which leads to no negative component in the total power curve. Moreover, as the intergroup oscillation is formed in the network under instantaneous stimulation, offsetting occurs if the phase difference is distinct.

## The relationship between neuron number and network energy features

### The relationship between the neuron number and the energy feature

**Figure 11:**
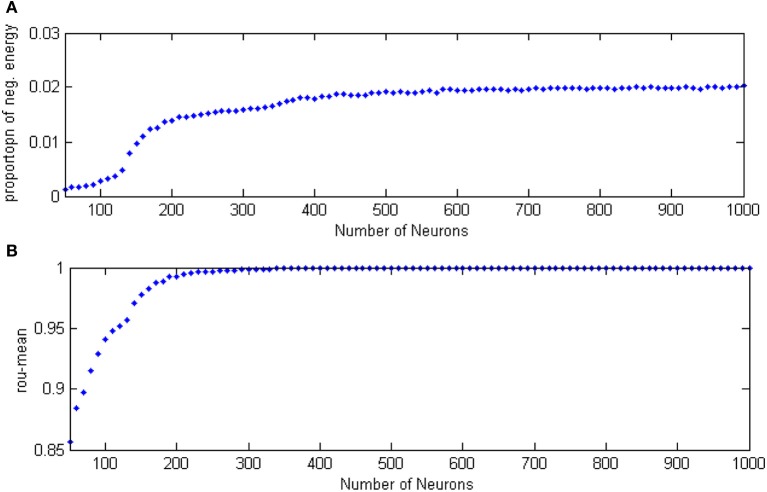
**(A)** The curve of α(*t*) varies as a function of the neuron amount. **(B)** The curve of ρ_mean_ varies as a function of the neuron amount.

For the study of the relationship between the neuron number and the energy feature, let the signal transform delay be uniformly distributed in [0.2 ms, 1.7 ms] (Dhamala et al., 2004) and the coupling strength is uniformly distributed in [0, 0.8]. The negative energy ratio α(*t*) and MCC are calculated as the neuron number varies from 50 to 1000, these are shown in Figures 11A,B.

Each point in Figure 11 was obtained as follows:

1. Let signal transform delay uniformly distribute in [0.2 ms, 1.7 ms] and the coupling strength uniformly distribute in [0, 0.8];
2. Stimulate any two neurons in the network at −40 mV for 0.1 ms;
3. Calculate the total power consumed by the overall neuron population *P*_*m*_(*t*);
4. Obtain α and ρ_mean_ by the way of Equations (8)–(12);
5. Repeat the process above 10 times and yield 10 of α and ρ_mean_ to obtain the average of these 10 α and ρ_mean_, termed α and ρ_mean_
6. α and ρ_mean_ are the values of vertical coordinates in Figures 10A,B.

**Figure 12:**
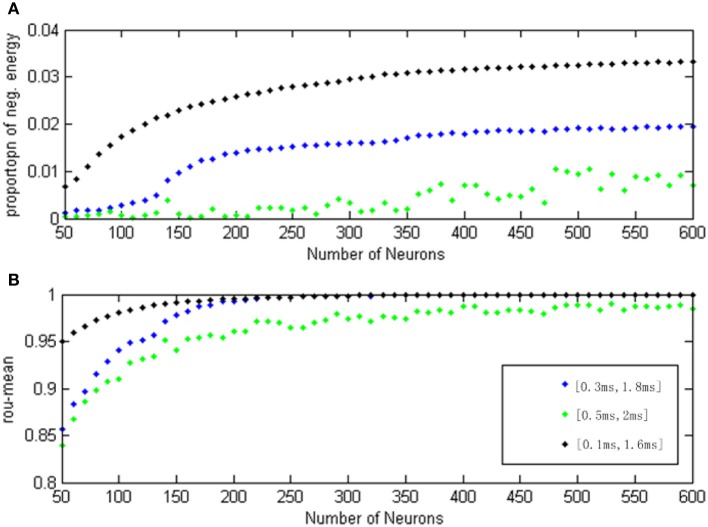
**(A)** The curve of α(*t*) varies as a function of the neuron amount for different transform delay distribution interval. **(B)** The curve of ρ_mean_ varies as a function of the neuron amount for different transform delay distribution interval.

According to Figure 11, the relationship between the neuron number and the negative energy is monotonic, which means as the number of neurons increases, the negative energy ratio also grows. When there are more than 400 neurons, “saturation” occurs, which is a consequence of the saturation of synchronization. In the mean time, the MCC showed a similar feature with the variation of the neuron amount. This indicates that the energy-based neural coding theory is also capable of representing network activity synchronization and is highly accordant with the traditional measure of correlation coefficient. In fact, energy-based neural coding is superior to correlation coefficient-base analysis. On one hand, the MCC curve comes to saturation when the neurons outnumber 200, whereas saturation occurred as the number of neurons exceeded 400 for the negative energy ratio. Moreover, the negative energy ratio maintained a slope in the saturation area. On the other hand, the superiority can be employed to study the influence of multiple variable parameters on synchronization, as Figure 12 shows.

In Figure 12, the signal transform delay is uniformly distributed in [0.1 ms, 1.6 ms] (black dot), [0.3 ms, 1.8 ms] (blue dot), and [0.5 ms, 2 ms] (green dot), and each point (both α(*t*) and ρ_mean_) is achieved in the same way as for Figure 11.

According to Figure 12, the negative energy ratio and MCC both reflected the differences of the neuron number and signal transform delay. When there were more than 200 neurons, however, the MCC curves with transform delay intervals of [0.1 ms, 1.6 ms] and [0.3 ms, 1.8 ms] are almost indistinguishable. Moreover, the MCC curve with transform delay uniformly distributing in [0.5 ms, 2 ms] is relatively close to the other two curves, which is a poor distinguishing feature for the variation of signal transform delay. In this sense, the correlation coefficient can merely reflect the variation for single parameter. Nevertheless, negative energy ratio can distinctly and simultaneously reflect the signal transform delay and the neuron number; the three curves did not converge. It can be easily noted that the negative energy power decreased as the distribution interval of signal transform delay moved away from zero.

According to the discussion above, the measure of energy can yield more valid, valuable conclusions dealing with mutually coupled neuron populations' synchronization and the parameter distribution. Moreover, it is capable of addressing many complex problems that traditional methods cannot.

### The relationship between neural energy and the coupling strength

**Figure 13:**
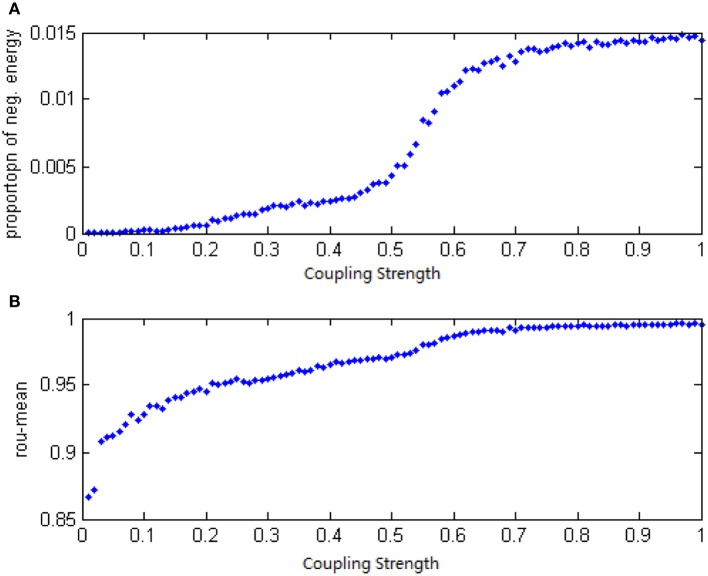
**(A)** The curve of α(*t*) over the variation of coupling strength distributing interval. **(B)** The curve of ρ_mean_ over the variation of coupling strength distributing interval.

Given a neural network with 200 neurons and the signal transform delay uniformly distributed in [0.3 ms, 1.8 ms], we studied the relationship between neural energy feature and the coupling strength. The total power consumed by the overall network was numerically calculated with varying coupling strength distributing intervals, and the negative ratio α(*t*) was obtained as well as the MCCs ρ_mean_. The curve of α(*t*) and ρ_mean_ over the variation of coupling strength distributing interval is plotted in Figures 13A,B.

Each point in Figure 13 is obtained as follows:

1. Let the coupling strength among neurons uniformly distribute in [0, 0.1 + Δ], where Δ is 0, 0.01, 0.02,…, 1, totally 100 value, and each Δ correspond to a point in the figure. There is respectively 100 points in Figures 13A,B.
2. Employ a neural network with 200 neurons and a signal transform delay uniformly distributed in [0.3 ms, 1.8 ms];
3. Stimulate any of two of the neurons in the network at −40 mV for 0.1 ms;
4. Calculate the total power consumed by the neuron population *P*_*m*_(*t*);
5. Calculate α and ρ_mean_ in the way of Equations (8)–(12)
  1. Repeat the process above 10 times to yield 10 α and ρ_mean_ and obtain the averages: α and ρ_mean_.
  2. α and ρ_mean_ are the vertical coordinate values in Figures 13A,B.

According to Figure 13, both the negative energy ratio and the MCC are monotonic with the variation of the coupling strength: the bigger value of the coupling strength is distributed, the bigger values of the negative ratio and the MCC yield. However, the changing of slopes is different: it is clear that the curve of negative energy ratio has two inflection points. Based on the analysis above, it is result in the asynchronous intergroup oscillation. That means, the energy-based neural coding reflects not only the synchronizing affection caused by coupling strength, but the change of the synchronization of intergroup oscillation as the coupling strength varies.

### The relationship between the signal transform delay and the energy feature

**Figure 14:**
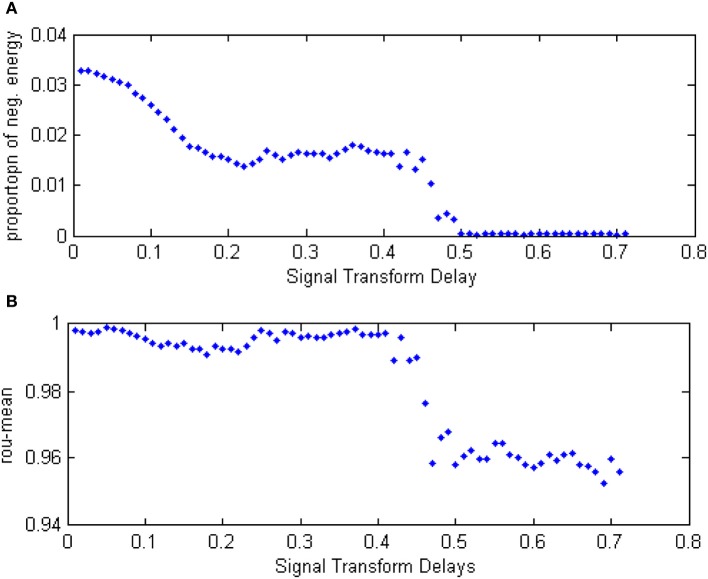
**(A)** The curve of α(*t*) over the variation of distributing interval of signal transform delay. **(B)** The curve of ρ_mean_ over the variation of distributing interval of signal transform delay.

In order to study the relationship between energy feature and the signal transform delay, a network with 300 neurons was employed, where the coupling strength was distributed in [0, 1]. The total power consumed was calculated under the instantaneous stimulation with various distributing interval of signal transform delay, and therefore the curve of negative energy ratio α(*t*), shown in Figure 14A was obtained.

Each point in Figure 14 was obtained as follows:

1. The signal transform delay is distributed in the interval set [0.01 + Δ, 1.5 + Δ], where Δ is 0, 0.01, 0.02,…, 0.7, respectively, for each interval and corresponds to one dot in Figure 14. There are a total of 70 dots in Figures 14A,B.
2. Let the neuron number be 300 and the coupling strength uniformly distributed in [0, 1].
3. Stimulate any two neurons in the network at −40 mV for 0.1 ms.
4. Calculate the total power consumed by the neuron population *P*_*m*_(*t*);
5. Calculate α and ρ_mean_ in the way of Equations (8)–(12)
6. Repeat the process above 10 times to yield 10 α and ρ_mean_ and obtain the averages: α and ρ_mean_.
7. α and ρ_mean_ are the vertical coordinate values in Figures 13A,B.

In Figure 14, the curve of the negative energy ratio and the MCC can be divided into four sections. The first corresponds to the horizontal axis value from 0 to 0.2. In this section, the negative energy ratio decreased as the interval of signal transform delay moved away from zero. The MCC, however, shows no such trend saliently because MMC cannot reflect intergroup oscillation synchronization, whereas the negative energy ratio can. The second section corresponds to the curve where the horizontal axis values range from 0.2 to 0.4. In this section, neither the negative energy ratio curve or the correlation coefficient prominently change as the distributing intervariation of the signal transform delay. It can be inferred that the signal transform delay only has a tiny influence on network activity synchronization. The third section corresponds to the horizontal axis values from 0.4 to 0.5. In this section, the negative energy curve and the correlation coefficient both dropped drastically. In the fourth section, the horizontal axis value is greater than 0.5, the negative energy ratio maintained near zero, while the correlation coefficient kept a relatively large value. This phenomenon illustrates that intergroup synchronization is faint, but the internal synchronization was well-sustained.

According to the analysis above, it can be seen that the relationship between the negative energy ratio and the signal transform delay is sectional. This analysis, combined with the correlation coefficient, shows that the synchronization of the network activity is discontinuously influenced by variation in the signal transform delay. Moreover, compared with the correlation coefficient, a traditional measure, more detailed information can be generated with energetical measurement.

### Energy features under multiple variable parameters

**Figure 15:**
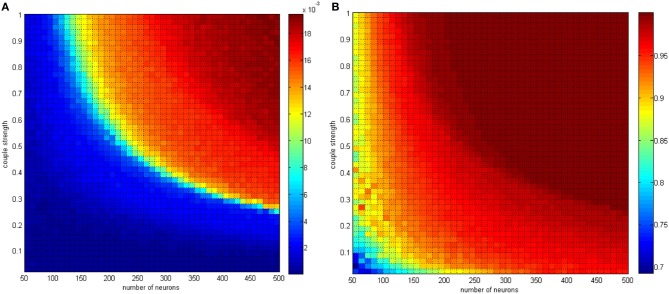
**(A)** The negative energy ratio as the neuron amount and the coupling strength simultaneously changes. **(B)** Correlation coefficient as the neuron amount and the coupling strength simultaneously changes.

**Figure 16:**
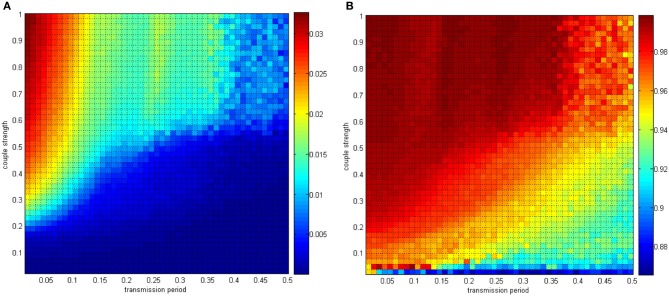
**(A)** The negative energy ratio as the coupling strength and the signal transform delay simultaneously changes. **(B)** The correlation coefficient as the coupling strength and the signal transform delay simultaneously changes.

The analysis of the relationship between the negative energy ratio and the correlation coefficient mainly concerned single variable parameters. The two-variable parameters circumstance is shown in Figures 15, 16.

In Figure 15, the neuron number changed gradually from 50 to 500, and the distributing interval of the coupling strength changed gradually from [0, 0.02] to [0, 1]. The signal transform delay was uniformly distributed in [0.3 ms, 1.8 ms]. For each parameter set, any two neurons are stimulated for 0.1 ms at −40 mV to force them fire, and we could determine the total power consumed in 30 ms and the membrane potential. Therefore, the negative energy ratio α and the correlation coefficient ρ_mean_ are obtained. Each point in Figure 15 is the mean value of 10 simulations.

According to the simulation results, it can be inferred that:

1. Increasing the neuron number and the coupling strength both lead to a negative energy ratio and an increasing correlation coefficient with a coherent trend. Considering the analysis of single-variable parameters, the effect of the neuron number and the coupling strength can be determined.
2. In Figure 14A the negative energy ratio gradually increased. However, in Figure 15A, the correlation coefficient enhanced drastically at first and then came to saturation as the horizontal and ordinate value increased. In other words, compared with traditional correlation coefficients, the quantitative measure of energy method possesses better hierarchy and better reflects the relationship between itself and the network parameters.

The conclusion above is consistent with the previous discussion. Multiple oscillation groups will be emerge under the instantaneous stimulation after the network achieves a stable state, and the intergroup oscillation is not always synchronous and is sensitive to the distributing interval parameter. It is hard to determine if this problem can be analyzed by the traditional correlation coefficient measure. However, with the energy method studied in this paper, a better effect can be achieved.

In Figures 15, 16, the distributing interval of the coupling strength changed gradually from [0, 0.02] to [0, 1], and the distributing interval of the coupling strength changed gradually from [0.01, 0.01 + 1.5] to [0.5, 0.5 + 1.5]. The number of neurons was maintained at 200. For each parameter set, any two neurons are stimulated for 0.1 ms at −40 mV to force them to fire; hence, the total power consumed in 30 ms is simulated, as well as the membrane potential. Therefore, the negative energy ratio α and the correlation coefficient ρ_mean_ are obtained. Each point in Figure 15 is a mean value of 10 simulations. It can be seen that a similar result was achieved.

**Figure 17:**
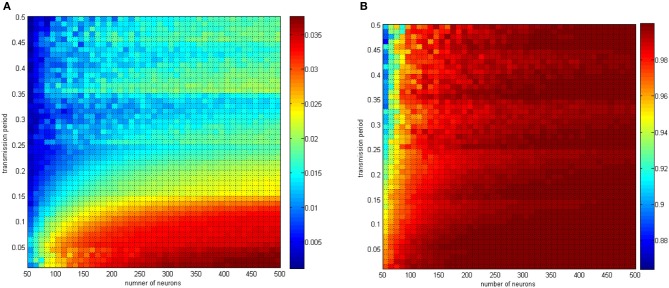
**(A)** The negative energy ratio as the coupling strength and the signal transform delay simultaneously changes. **(B)** The correlation coefficient as the coupling strength and the signal transform delay simultaneously changes.

In Figure 17, the distributing interval of the coupling strength changed gradually from [0, 0.02] to [0, 1] and the neuron number changed gradually from 50 to 500. The coupling strength was uniformly distributing in [0, 1]. For each parameter set, any two neurons are stimulated for 0.1 ms at −40 mV to force them fire, and the total power consumed in 30 ms and the membrane potential are simulated. Therefore, the negative energy ratio α and the correlation coefficient ρ_mean_ are obtained. Each point in Figure 15 is the mean value of 10 simulations.

With the calculation in Figure 17, the discussion about signal transform delay can be further explained. Figure 17B shows that signal transform delay has a small influence on MCC but has a distinct effect on the negative energy ratio. This is because the negative energy ratio reflects the synchronization activity to a great extent, which the MCC is rarely capable of. Hence, we found that the intergroup asynchronization was mainly due to long signal transform delay.

## Conclusions

In this paper, we studied the relationships among neural energy features, network parameters, and oscillating characteristics. What should be stressed is that the value of a parameter can vary from one specific function to another, but the relationships remain coherent. Specifically, they are:

1. The relation between negative energy ratio and the amount of neurons is monotonic increasing, as well as the synchronous oscillation (measured by correlation coefficient).
2. The relation between negative energy ratio and the coupling strength is monotonic increasing, as well as the synchronous oscillation (measured by correlation coefficient).
3. The relation between negative energy ratio and the time delay of transmitting signal is monotonic decreasing, as well as the synchronous oscillation (measured by correlation coefficient).
4. Negative ratio can better represents the variation of synchronous activity due to the multiply changed parameters.
5. Negative ratio can better represents the intergroup asynchronization.

The study method proposed in this paper represent the networks' parameters and its activity with respect to the energy method. It is a novel method rather than a precise conclusion. But still, it can quantitatively represent the status of oscillation with respect to synchronization or asynchronization. Multiple variable parameters will be an important focus of further research.

Though the networks studied in this paper is structural, it is an important foundation for further development for energy method to address on functional networks, which is closely related to cognitive and behavior problems. And this is another focus in the future.

The energy method is a new theory to investigate the global behavior of brain activity. One significant characteristic is the superposition property. In experimental neuroscience, recording all neural impulses, even in a small encephalic region, is virtually impossible. However, it is possible to measure the total energy of the region. Based on this, the study method proposed in this paper is a effective tool for experimental neuroscience; parameter distribution of a given encephalic region can be estimated by the energy method proposed in this paper. This method can also be used to study differences between encephalic regions. It is of more practical value and promotive value compared with traditional numerical analysis.

The theoretical study of nervous system coding and decoding is in bottleneck period, and new methods are needed to augment traditional ones and achieve more valuable results. It is important to mention that the energy coding theory is in its infancy, and there are numerous problems that remain to be solved. However, the energy method has significant potential and could be useful for addressing questions that traditional neuroscientific methods have been unable to elucidate.

### Conflict of interest statement

The authors declare that the research was conducted in the absence of any commercial or financial relationships that could be construed as a potential conflict of interest.
